# Increase the amyloid pathology in meningitis‐induced cognitive impairment: sex differences

**DOI:** 10.1002/alz.089993

**Published:** 2025-01-03

**Authors:** Lucineia Danielski, Vijayasree V Giridharan, Ngozi Iwunze, Celso Catumbela, Rodrigo Morales, Tatiana Barichello

**Affiliations:** ^1^ The University of Texas Health Science Center at Houston, Houston, TX USA; ^2^ Universidade do Extremo Sul Catarinense, Criciuma, SC Brazil

## Abstract

**Background:**

Pneumococcal meningitis is a type of meningitis that may face long‐term neurological complications, leading to the hypothesis that it might contribute to the deposition of beta‐amyloid (Aβ) and predispose individuals to Alzheimer’s pathology.

**Method:**

Male and female APP/PS1 mice, 50 days old, were divided into control (n = 5) and meningitis (n = 6). Under anesthesia, an intracisternal injection of either artificial cerebrospinal fluid (CSF) as a placebo or 5 × 10^9^ colony‐forming units (CFU) of *S. pneumoniae* suspension was injected. Meningitis was confirmed through culture incubation. The Open Field Test (OFT) and Novel Object Recognition (NOR) tasks were conducted when the mice reached 180 days of age; after this, the hippocampus and prefrontal cortex (PFC) were stained to be evaluated by immunofluorescence.

**Result:**

Mice of both sexes experiencing meningitis displayed cognitive impairment (p < 0,001) evaluated for NOR, but the distribution of Aβ burden differed between males and females. Males exhibited elevated Aβ predominantly in the hippocampus (p < 0,05), while females showed heightened Aβ levels exclusively in the PFC (p < 0,05). Interestingly, only females with meningitis showed increased levels of IBA‐1, a marker for microglial reactivity, in both the hippocampus (p = 0,0048) and PFC (p = 0,0023) compared to their controls.

**Conclusion:**

The distinct patterns observed in Aβ distribution and microglial activation between sexes suggest a potential association, indicating that meningitis can induce elevated Aβ deposition, with microglia potentially playing a role in this divergent response.

